# A Systematic Review of the Effect of PTSD and Trauma on Treatment Outcomes for Eating Disorders

**DOI:** 10.1177/15248380231167399

**Published:** 2023-04-26

**Authors:** Sinead Day, Phillipa Hay, Wadad. Kathy Tannous, Scott J. Fatt, Deborah Mitchison

**Affiliations:** 1 Translational Health Research Institute, Western Sydney University, Penrith, NSW, Australia; 2 Translational Health Research Institute, School of Medicine, Western Sydney University, Penrith, NSW, Australia; 3 Mental Health Services Camden and Campbelltown Hospitals, South West Sydney Local Health District, NSW, Australia; 4 Translational Health Research Institute, School of Business, Western Sydney University, Penrith, NSW, Australia

**Keywords:** eating disorders, trauma, PTSD, treatment, outcomes

## Abstract

There is growing evidence of prior experiences of trauma and trauma-related symptoms among people with eating disorders; however, there is little understanding as to how post-traumatic stress disorder (PTSD) and exposure to traumatic events affect treatment outcomes. Without this knowledge, eating disorder clinicians are unable to tailor treatment to ensure good outcomes for the large percentage of this population that is affected by PTSD and trauma. This systematic review aimed to identify how PTSD and trauma exposure influence outcomes in eating disorder treatment. Systematic searches of PsycINFO, MEDLINE, PubMed, and Scopus databases identified 16 articles that met the inclusion criteria. The results indicated a negative effect on rates of eating disorder treatment completion and eating disorder psychopathology posttreatment. These findings were evident across studies that investigated the impact of a history of traumatic events as well as studies that investigated the impact of the presence of trauma-related symptoms seen in PTSD. Several methodological limitations were identified in the literature. These include: heterogeneous and unstandardized measures of PTSD and trauma, high attrition rates with follow-up, and insufficient data to enable comparisons by treatment setting, diagnostic presentation, and type of trauma exposure. The findings of this review have implications for future research and clinical care, including the importance of considering PTSD and trauma in assessment, treatment planning, and provision of both trauma-informed care and trauma-focused treatments for individuals with eating disorders.

Eating disorders are a complex and often difficult to treat category of mental illness. They are associated with high rates of mortality and disability (van Hoeken & Hoek, 2020) and typically have a chronic course, with long-term remission rates ranging from 30% to 63% for anorexia nervosa (AN) (Eddy et al., 2017; Fichter et al., 2017) and 40%−75% for bulimia nervosa (BN) (Keel et al., 2010; Steinhausen & Weber, 2009). Given that the rates of long-term improvement remain modest, there has been increasing research on moderators and predictors of treatment outcomes, as these may lead to treatment innovations and improvements. Current treatment approaches for eating disorders include psychological therapies such as cognitive behavioral therapy (CBT), delivered in a variety of settings such as inpatient hospitalization, hospital day programs, and regular outpatient consultation with private clinical and allied health professionals. A meta-analysis of 126 studies of individuals receiving eating disorder treatment identified that predictors of better outcomes included greater motivation and improvement in symptoms early in treatment and fewer comorbidities (Vall & Wade, 2015). Comorbidities are very common among individuals with eating disorders, with one narrative review finding that over 70% of people with eating disorders report a co-occurring psychiatric disorder (Keski-Rahkonen & Mustelin, 2016). The potentially detrimental effect of comorbidities on eating disorder treatment outcomes has been supported in recent findings. For example, comorbid depression and anxiety have been found to predict greater eating disorder psychopathology at discharge from residential and partial hospitalization (Fewell et al., 2017) and from outpatient CBT for binge eating disorder (BED) (Lydecker & Grilo, 2021).

Another class of common comorbidity among eating disorder populations includes trauma-related disorders. Trauma is defined, typically, as involving an intense emotional and biological stress response to a non-ordinary event that is experienced as threatening or aversive (Dalenberg et al., 2017). The Substance Abuse and Mental Health Services Administration (SAMHSA, 2014) similarly conceptualizes trauma in relation to the three “E”s—the event(s), the individual’s experience of the event(), and the lasting mental, physical, emotional, and social effects. However, as studies vary as to whether they examine individuals’ history of exposure to traumatic events or its effects, these are discussed separately throughout.

## Post-Traumatic Stress Disorder and Eating Disorders

These lasting effects of traumatic events are most commonly, clinically captured by the diagnosis of post-traumatic stress disorder (PTSD). The symptoms of PTSD include intrusive memories, flashbacks, and distress occurring in relation to being exposed to actual or threatened death, serious injury, or sexual violence (Diagnostic and Statistical Manual of Mental Disorders, fifth edition [DSM-5]; American Psychiatric Association, 2013). In a sample of 107 female patients receiving inpatient or outpatient treatment for an eating disorder, the diagnosis of PTSD was found in 23% of those with AN and 26% of those with BN (Tagay et al., 2014). Similarly, among 642 adults (96.7% female) receiving residential treatment for an eating disorder, 49% were found to meet the criteria for PTSD (Brewerton et al., 2020). In a broader synthesis of 33 eating disorder samples, the pooled prevalence of PTSD was 24.6% when weighted by study quality (Ferrell et al., 2022). A recent systematic review on comorbid PTSD in individuals with eating disorders noted that maladaptive emotion regulation may act as the mediating mechanism (Rijkers et al., 2019). As such, eating disorder behaviors are theorized to enable the avoidance of trauma-related thoughts/feelings and reduce hyperarousal, which are common symptoms of PTSD (Trottier & MacDonald, 2017).

The impact of trauma is important to consider regardless of whether the person who experienced the traumatic event meets the full criteria for a diagnosis such as PTSD. It is well established that the trauma response lies on a continuum, and that even subthreshold PTSD has been found to be associated with significant impairment and depression (Cukor et al., 2010). Subthreshold PTSD has been suggested as a risk factor for eating disorders, particularly for symptoms of BN (Brewerton, 2007), which was highlighted by subsequent findings that its prevalence rate was 47.3% for women and 66.2% for men in a sample of individuals with BN (Mitchell et al., 2012). As such, trauma and PTSD are prevalent and potentially impactful for eating disorders, regardless of whether criteria are met for a diagnosis of PTSD.

## Traumatic Events and Eating Disorders

Even subthreshold PTSD status, however, may not capture the effects of all forms of trauma. The effect of trauma on eating disorders has also been examined at the level of trauma history, rather than whether the diagnostic criteria are met for PTSD. Prevalence rates of prior traumatic experiences have been found to exceed 90% in an inpatient and outpatient sample of individuals with AN and BN using a standardized measure of common traumatic events (Tagay et al., 2014). Similarly, a study of over 5,000 participants from the National Comorbidity Survey-Replication found that both men and women with eating disorders had experienced most forms of traumatic events at a significantly higher rate than the general population, particularly for interpersonal traumas such as sexual assault (Mitchell et al., 2012).

Prolonged interpersonal trauma, often occurring in childhood, has been highlighted among eating disorder epidemiological literature. Studies conducted in inpatient, residential, or partial hospitalization settings for eating disorder treatment have found that participants report at least two adverse childhood experiences (ACEs) on average, with approximately one in four people reporting four or more experiences (Rienecke et al., 2021). ACEs refer to childhood experiences with potentially detrimental consequences for future health outcomes, with such experiences including verbal, physical, and sexual abuse; neglect; and household dysfunction (e.g., parental divorce, domestic violence) (Felitti et al., 1998). A review of literature on the epidemiological relationship between trauma and eating disorders highlighted a clear association between trauma exposure, particularly in childhood, and eating disorders (Trottier & MacDonald, 2017). Traumatic events, such as childhood abuse, have therefore been increasingly recognized as being more prevalent among eating disorder populations.

## Effects on Eating Disorder Severity

PTSD and exposure to traumatic experiences have both been consistently shown to be associated with more severe eating disorder pathology. With regards to trauma history, Trottier and MacDonald (2017) found that childhood abuse is associated with earlier age of eating disorder onset and more severe eating disorder symptoms. They posited that mediating mechanisms likely include emotion dysregulation, dissociation, and maladaptive core beliefs (e.g., about defectiveness and abandonment). Meta-analytic findings similarly indicated that childhood maltreatment has a dose–response relationship with eating disorder illness severity (Molendijk et al., 2017). Comorbid PTSD has also been found to be associated with more severe eating disorder pathology in multiple studies of individuals on admission to hospital inpatient and outpatient, and residential treatment settings, compared to those without PTSD (Brewerton et al., 2020, 2021; Rijkers et al., 2019; Scharff et al., 2019). Thus, both PTSD and a history of traumatic experiences appear to correlate with eating disorder prevalence and symptom severity, with some evidence for dose-dependent effects with greater exposure to trauma.

Although the varying usages of the term “trauma” (as both event and effect) thus complicate the understanding of its relationship with eating disorders, both exposure to traumatic events and the presence of PTSD symptoms appear prevalent among individuals with eating disorders (Brewerton et al., 2020; Tagay et al., 2014). There has been considerable research on how trauma and PTSD influence the development and maintenance of eating disorders (Trottier & MacDonald, 2017), as well as their association with more severe eating disorder symptoms (e.g., Brewerton et al., 2020; Molendijk et al., 2017; Rijkers et al., 2019; Scharff et al., 2019). However, relatively less attention has been given to how trauma and PTSD impact treatment outcomes. Given the generally modest outcomes from the available eating disorder treatments, it is important to clarify the current state of research, including gaps, on whether and how trauma and PTSD could affect the likelihood of someone completing and benefitting from eating disorder treatment. Such information is important in guiding clinical innovations and decision-making about whether eating disorder treatments require tailoring, or adjunct interventions for individuals who have experienced trauma and PTSD, and whether eating disorder clinicians require more comprehensive training in trauma-informed care.

## Current Review

In sum, current research indicates that (1) prior traumatic events and current trauma-related diagnoses such as PTSD are highly prevalent among individuals with eating disorders; (2) trauma and PTSD are associated with more severe eating disorder symptoms; and (3) current rates of improvement with eating disorder treatment are limited by factors including comorbid psychopathology. Despite this, to our knowledge there have been no prior reviews of the impact of trauma and PTSD on eating disorder treatment outcomes. Our aim is to provide a systematic review of how trauma history and symptoms of PTSD influence the effectiveness of eating disorder treatment in reducing eating disorder symptoms and to further the understanding of who is more or less likely to benefit from eating disorder treatment.

## Method

### Databases, Search Terms, and Search Strategy

**Figure 1.**
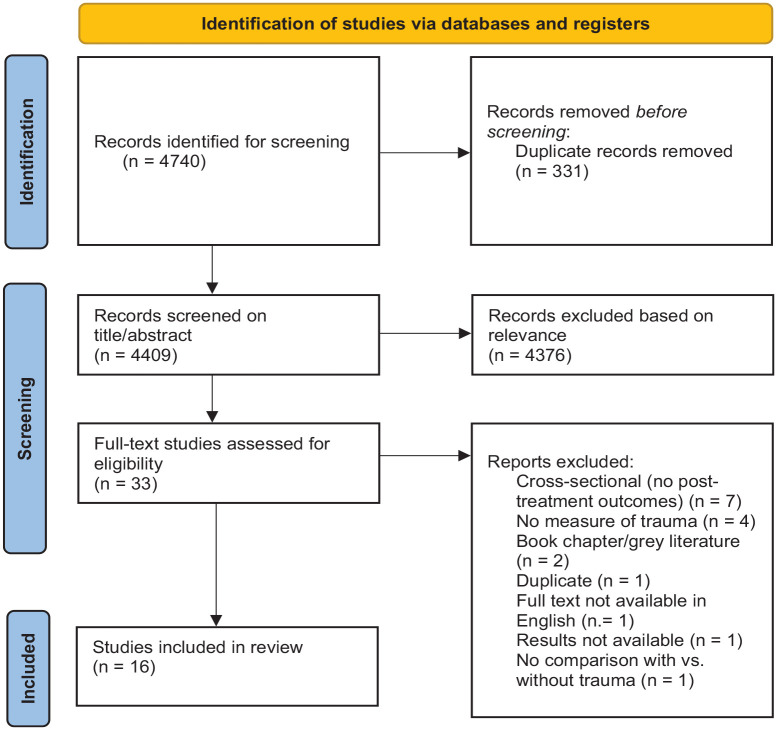
PRISMA diagram of systematic review search.

The protocol for this review was registered with the International Prospective Register of Systematic Reviews (PROSPERO; https://www.crd.york.ac.uk/prospero, registration number CRD42022302872). There was only one update made post-registration, which was to clarify the language used in the study selection criteria regarding the need for studies to have examined the relationship between trauma history or PTSD and eating disorder-related outcomes. This update was published at the link above. The Preferred Reporting Items for Systematic Reviews and Meta-Analyses (PRISMA) guidelines (Shamseer et al., 2015) were used in designing the review protocol (see Figure 1). Searches were conducted on May 10, 2022, using the PsycINFO, PubMed, MEDLINE, and Scopus databases. All studies published prior to the search date were screened for eligibility. Studies were searched for using the following terms: (“*eating disorder*” OR “*disordered eating*” OR “*anorexia*” OR “*bulimia*” OR “*binge eating*” OR “*arfid*”) AND (“*trauma*” OR “*post-trauma*” OR “*PTSD*” OR *“CPTSD*” OR *“adverse childhood experiences*” OR “*ACE*” OR “*child maltreatment*” OR “*abuse*” OR “*neglect*” OR “*adverse life events*”) AND (“treatment”) AND (“*moderator*” OR “*moderate*” OR “*predictor*” OR “*predict*” OR “*mediator*” OR “*mediate*”). These search terms were derived from previous reviews in similar areas (e.g., Rijkers et al., 2019; Trottier & MacDonald, 2017). All studies including these terms were then assessed for relevance.

### Eligibility Criteria

The inclusion criteria used were as follows: (1) study published in English; (2) quantitative study or mixed methods study from which quantitative data can be extracted; (3) sample includes participants undergoing psychological treatment for eating disorders; (4) study reports participants’ history of trauma, PTSD or complex PTSD (CPTSD) diagnoses, or other trauma-related symptoms based on standardized self-report or interviewer-administered assessments; (5) study includes measure of psychological and/or behavioral treatment outcomes related to eating disorder psychopathology; and (7) study compares eating disorder treatment outcomes between individuals with and without trauma history and/or symptoms. Studies were excluded based on the following criteria: (1) heterogeneous sample from which it is not feasible to discern which participants undergoing eating disorder treatment reported a history of trauma, trauma-related symptoms, or a PTSD/CPTSD diagnosis; (2) study did not report change in eating disorder symptoms as a treatment outcome; (3) review articles or meta-analyses; (4) book chapters, reviews, conference papers, case studies, dissertations/theses, commentaries/editorials, notes, and guidelines; and (5) full text unavailable.

### Selection Process

As depicted in Figure 1, from the initial 4,740 records identified, 16 articles were included in the review. The online tool Covidence (Covidence systematic review software, Veritas Health Innovation, Melbourne, Australia) was used in the screening and quality assessment processes. Titles and abstracts were independently screened by the lead author (SD) and a second reviewer (SF) to identify studies that potentially met the eligibility criteria. Any discrepancies were discussed between reviewers until consensus was met, with any unresolved discrepancies arbitrated by a third reviewer (DM). After the initial screening, the full texts of the identified studies were retrieved and assessed by the lead author to determine eligibility. A third of the studies at the full-text review stage (11 articles) were independently assessed by the second reviewer and no discrepancies were found. After articles meeting the criteria for inclusion were determined at the full-text stage, the lead author extracted relevant data and administered the quality assessment tool.

Data were synthesized in table format according to treatment setting, measure of eating disorder symptoms (e.g., remission status, change in global eating disorder psychopathology, change in eating disorder behavior frequency), measure of trauma (e.g., trauma history, diagnosis of PTSD/CPTSD, score on trauma symptom questionnaire), and outcomes (including strength of effect). Demographic characteristics—including sample size, study location, age, gender, and eating disorder diagnostic groups—were tabulated separately. Where information was missing, the corresponding author of the relevant study was contacted and requested to provide this information.

### Assessment of Study Quality

The Effective Public Health Practice Project (EPHPP; Thomas et al., 2004) quality assessment tool for quantitative studies was used to evaluate the study quality. This tool provides a rating of study quality across several factors including: selection bias, study design, confounders, blinding, data collection method, and withdrawals and dropouts. These categories include criteria to rate them as “strong,” “moderate,” or “weak,” with the total number of each rating determining the global rating for the study. The tool was found to have fair inter-rater agreement for individual domains and excellent agreement for the final grade in a systematic review of 20 randomized controlled trials (Armijo-Olivo et al., 2012). In the current review, all of the articles were scored by the lead author and five of the articles were also independently scored by a second author (SF) and no discrepancies were found.

## Results

### Study Design and Sample Characteristics

Demographics and study design are reported in Table 1. All studies were conducted in developed Western countries, with six conducted in the United States, three in Italy, two in the United Kingdom, two in Canada, and one each in Norway, Belgium, and Germany. Most participants were female—nine studies only included female participants and seven studies had mixed gender samples, in which most (82.1%−98.6%) participants still identified as female. Although ethnicity was not reported by the nine studies, in the remaining seven studies the majority of participants identified as White/Caucasian.

**Table 1.** Demographic Characteristics of the Studies Included in Systematic Review.

Study	Location	*N*	Gender	Age Range, Mean Age (SD)	Ethnicity	Eating Disorder Diagnoses	Length of Treatment	Quality Assessment Rating (EPHPP)
Anderson et al. (1997)	United States	74	Female	[n.r.], 27.0 (9.3)	89% Caucasian	67.6% BN, 4.1% AN-BP, 9.5% AN with BN history, 12.2% both AN and BN, 6.8% BED	n.r.	Moderate
Carter et al. (2006)	Canada	77	Female	[n.r.], 25.5 (7.8)	93% Caucasian, 3% Asian, 4% African Canadian or East Indian	43% AN-BP, 57% AN-R	Mean length 12.4 weeks	Weak
Cassioli et al. (2022)	Italy	120	Female	[n.r.], 25.22 (9.55)	n.r.	AN	Median 42 sessions	Strong
Castellini et al. (2020)	Italy	50	Female	[n.r.], 24.60 (7.01) for AN-R, 23.40 (6.20) for AN-BP	n.r.	30% AN-R, 70% AN-BP	Minimum 40 weeks	Moderate
Fichter et al. (2008)	Germany	264	Female	[n.r.], 29.3 (8.4) for BED, 25.6 (6.7) for BN-P	n.r.	25.8% BED, 74.2% BN-P	Mean 76.7 days BED, 95.5 days BN-P	Weak
Hazzard et al. (2021)	United States	112	82.1% female, 17.9% male	[18–64], 39.7 (*13.4*)	91.1% Caucasian	BED	17 weeks	Moderate
Mahon, Bradley et al. (2001)	United Kingdom	114	Female	[n.r.], 26.7 (7.6)	n.r.	BN	n.r.	Weak
Mahon, Winston et al. (2001)	United Kingdom	111	Female	[n.r.], 24.47 (5.9)	n.r.	BN	n.r.	Weak
Mensinger (2021)	United States	70	97.1% female, 0% male, 1.4% other	[26–68], 45.5 (*10.9*)	87.1% White, 5.7% Hispanic, 4.3% mixed race, 1.4% other	47.2% BED, 30.6% OSFED, 11.1% BN, 11.1% AN	6 weeks	Weak
Mitchell et al. (2021)	United States	2809	Female	[13–62], 25.14 (10.99)	80.5% White, 2.1% Black, 2.3% Asian or Pacific Islander, 0.5% Native American, 3.5% multiracial, 2.2% other, 6.1% Latinx	24.0% AN-R, 19.2% AN-BP, 27.2% BN, 15.8% OSFED, 5.4% BED, 6.5% atypical AN, 1.6% ARFID	Mean 32.1 days	Weak
Pingani et al. (2012)	Italy	186	93.5% female, 6.5% male	[16–67]^a^, 27.6 (8.5)	Italian^a^	14.5% AN, 29% BN, 56.5% EDNOS	Mean 75.7 days of inpatient, 41.4 days of day hospital	Weak
Rienecke et al. (2022)	United States	1819	88.5% female, 9.9% male, 0.8% nonbinary, 0.6% transgender, 0.4% undisclosed	[n.r.], 26.86 (9.82)	n.r.	30.4% AN-R, 28.4% AN-BP, 13.2% BN, 9.7% ARFID, 5.7% BED, 12.6% OSFED	n.r.	Moderate
Scharff et al. (2021)	United States	1055	Female	[13–75], 24.73 (10.72)	80.9% White	42.7% AN, 28.9% BN, 22.3% OSFED	Mean 33.3 days	Moderate
Serra et al. (2020)	Belgium	142	88% female, 12% male	[20–64]^a^, 38.65 (10.83)	Caucasian^a^	BED	6 months	Strong
Trottier (2020)	Canada	151	94.7% female, 4.6% male, 0.7% transgender	[n.r.], 28.1 (8.6)	n.r.	66.2% BN, 33.8% OSFED	n.r.	Strong
Vrabel et al. (2010)	Norway	74	98.6% female, 1.4% male	[n.r.], 29.0 (7.3)	Caucasian	13 (17.6%) AN, 37 (50%) BN, 24 (32.4% EDNOS)	22–23 weeks AN, 15 weeks BN	Moderate

*Note.* n.r. = not reported. AN = anorexia nervosa; AN-BP = anorexia nervosa, binge-purge subtype; AN-R = anorexia nervosa, restrictive subtype; ARFID = avoidant/restrictive food intake disorder; BED = binge eating disorder; BN = bulimia nervosa; EDNOS = eating disorder not otherwise specified; EPHPP = effective public health practice project quality assessment tool for quantitative studies (Thomas et al., 2004); OSFED = other specified feeding and eating disorder.

a Information provided upon request from study author.

Studies reflected a variety of eating disorder diagnoses. Eleven studies included a mixed sample of multiple eating disorder diagnoses, while two studies each included only participants with BED (Hazzard et al., 2021; Serra et al., 2020) or BN (Mahon, Bradley, et al., 2001; Mahon, Winston, et al., 2001), and one study’s sample was limited to individuals with AN (Cassioli et al., 2022). There was similar heterogeneity in treatment setting as follows: four studies were conducted in inpatient settings, seven in outpatient, two residential, one used online guided self-help, and two included multiple treatment settings. Although the description of the treatment approach was often sparse, most studies appeared to use CBT as the primary psychological treatment, with some also including adjunct approaches such as nutritional rehabilitation.

In relation to study design, most studies used repeated measures designs comparing outcomes from pre to posttreatment for a single intervention. Several studies included follow-up, ranging in duration from 3 months to 12 years. Ten out of 16 studies reported controlling for covariates in their relevant statistical analyses. Most commonly, these included demographic characteristics of age and body mass index (BMI), as well as baseline eating disorder psychopathology. As expected, there was variation in whether studies examined trauma exposure or PTSD symptoms, and in what measurement the tools were used. Twelve studies measured history of traumatic events, with eight of these specifically assessing childhood trauma history. Four studies assessed PTSD diagnosis and one study by Mensinger (2021) measured traumatic stress as another indicator of trauma-related symptoms but did so using a common self-report PTSD diagnostic tool. The results of the articles are reported below, organized by treatment outcome, and distinguished between trauma exposure and trauma-related symptoms (including PTSD). Findings and effect sizes are also summarized in Table 2. Effect sizes were available for 9 of the 16 studies and ranged in magnitude from small to large.

**Table 2.** Results of Studies Included in Systematic Review.

Study	Treatment Setting	Eating Disorder Treatment Outcome (Measure)	Trauma Outcome (Measure)	Results	Covariates	Effect Size
Anderson et al. (1997)	Inpatient (group, individual and family therapy, nutritional rehabilitation, medication)	Eating disorder symptoms (EDI-2; nurse-reported frequency of eating disorder behaviors [meal refusal, ritualistic eating, and exercise])	History of sexual abuse (structured interview, author-derived)	No significant differences in degree of change in ED symptoms from pretreatment to 3-month follow-up. Those with a history of abuse had lower abstinence from ED behaviors than non-abused participants at follow-up, and were more likely to be hospitalized between discharge and follow-up (*d* = 0.75).		Medium
Carter et al. (2006)	Inpatient (group psychotherapy)	Time to discharge, premature dropout, and BMI	History of CSA (structured interview, author-derived)	No difference in mean time to discharge from treatment, rate of premature dropout, and mean BMI at discharge for those with and without a history of child sexual abuse. Earlier treatment termination for abused individuals with AN-BP versus AN-R or no trauma history.	Adult sexual abuse history	n.r.
Cassioli et al. (2022)	Outpatient (CBT-E)	Eating disorder symptoms (EDE-Q)	Childhood trauma history (CTQ)	Childhood trauma predicted reduced treatment efficacy for eating disorder symptoms at 1-year follow-up, mediated by higher baseline emotion dysregulation. Direct effect nonsignificant.	Age, BMI	n.r.
Castellini et al. (2020)	Hospital outpatient (CBT-E)	Resumption of menses (self-report)	History of emotional, physical, or sexual abuse (semi-structured interview)	History of childhood abuse predicted greater likelihood of and shorter mean time to resumption of menses.	Age, BMI	n.r.
Fichter et al. (2008)	Inpatient (multimodal, including CBT)	Eating disorder diagnosis (SIAB)	History of sexual abuse (semi-structured interview)	History of violent and severe sexual abuse and severity of sexual abuse each predicted poorer diagnostic outcome (still meeting ED criteria) at 12-year follow-up for BED (*d* = 0.58) but not BN-P.		Medium
Hazzard et al. (2021)	Outpatient (ICAT or guided self-help CBT)	Eating disorder symptoms (EDE)	Lifetime PTSD diagnosis (SCID-IV), history of childhood abuse (CTQ)	PTSD diagnosis predicted greater frequency of objective binge eating episodes at end-of-treatment (SMD = 1.99)^a^. History of moderate or severe childhood abuse predicted greater frequency of objective binge eating episodes at 6-month follow-up (SMD = 1.49)^a^, moderated by PTSD diagnosis (childhood abuse history was a significant predictor for those with lifetime PTSD but not those without). No effect of PTSD or childhood abuse history on global ED psychopathology.	Baseline frequency of objective binge eating episodes, age, gender, ethnicity, education, study site, and treatment group	Large
Mahon, Bradley et al. (2001)	Hospital outpatient (individual CBT or IPT)	Treatment dropout	History of CSA, physical abuse, or parental losses (semi-structured interview)	Greater number of traumatic events in childhood predicted greater likelihood of treatment dropout (*d* = −0.45).		Small
Mahon, Winston et al. (2001)	Hospital outpatient (psychotherapy and dietetic support)	Treatment dropout	History of CSA, physical abuse, and parental losses (interview)	Childhood trauma was associated with greater likelihood of dropout from treatment with a dose–effect response (*d* = −0.41).		Small
Mensinger (2021)	Online guided self-help (weight neutral self-care, intuitive eating, self-compassion)	Eating concerns (EDE-Q), binge eating episodes and overvaluation of shape/weight (QWEP-5)	Traumatic stress (PCL-5)	Reduction in traumatic stress mediated improvement in eating concerns, binge episodes, and overvaluation of shape/weight at posttreatment.	Age	n.r.
Mitchell et al. (2021)	Residential (Unified Treatment Model)	Eating disorder symptoms (EDE-Q)	PTSD diagnosis (clinical interview)	No association between PTSD diagnosis and different trajectory of ED symptom change (from admission to discharge or discharge to 6-month follow-up), treatment dropout, or clinically significant change from admission to discharge or 6-month follow-up.	Comorbid diagnoses, BMI, age, illness duration, education, and ethnicity	Not significant
Pingani et al. (2012)	Inpatient and hospital outpatient (CBT)	Treatment dropout	Childhood trauma history (EDQ)	Dropouts had significantly more traumatic experiences than treatment completers (OR = 2.35)^a^.		Small
Rienecke et al. (2022)	Inpatient, residential, and partial hospitalization (family therapy, dietetic support, and individual therapy using ACT, DBT, and ERP)	Eating disorder symptoms (EPSI)	Childhood trauma history (ACEs)	Aggregated across diagnoses, greater number of childhood trauma experiences was associated with higher rates of binge eating at end-of-treatment as both continuous and dichotomous predictor (for those with severe history of childhood trauma), but not with purging or restriction. Between diagnoses, at discharge childhood trauma was associated with greater purging for AN-R and BED.	Admission eating disorder scores, age, gender, diagnosis	n.r.
Scharff et al. (2021)	Residential (Renfrew UTM, individual and group psychotherapy)	Eating disorder symptoms (EDE-Q)	PTSD diagnosis (semi-structured interview)	PTSD diagnosis moderated change in symptoms from admission to discharge, with greater improvement for those with PTSD versus without. PTSD diagnosis predicted greater symptom recurrence at 6-month follow-up (*d* = 0.39).	BMI, age, and ED diagnosis	Small
Serra et al. (2020)	Hospital outpatient (group CBT)	Binge frequency (self-reported)	Trauma history (TEC)	Greater impact of traumatic experiences was associated with lower likelihood of remission from BED symptoms posttreatment (OR = 0.96). No effect of number of traumatic experiences on reduction in or remission from binges.	Baseline number of binges per week, baseline depression score, and dropout	Small
Trottier (2020)	Hospital outpatient (group CBT)	Treatment dropout	Current PTSD diagnosis (PCL-5)	PTSD diagnosis predicted greater likelihood of premature treatment termination (2.32 × greater risk) (*d* = 0.61).	Pretreatment depression, eating disorder psychopathology, binge and purge frequency, and clinical impairment	Medium
Vrabel et al. (2010)	Inpatient (CBT and ERP)	Eating disorder symptoms (EDE)	History of CSA (self-reported in medical chart)	Childhood sexual abuse predicted less improvement in eating disorder symptoms at 5 years posttreatment.		n.r.

*Note.* Effect sizes are shown as reported in each study. Where effect sizes were not reported, an effect size was calculated based on available information as Cohen’s *d* (Lenhard & Lenhard, 2016) or authors were contacted to request the effect size. The magnitude of effect sizes is interpreted using reported intervals for odd ratios (OR; Chen et al., 2010), Cohen’s *d* (Cohen, 1988), and standardized mean difference (SMD; Faraone, 2008). n.r. = not reported; ACEs = adverse childhood experiences questionnaire (Felitti et al., 1998); ACT = acceptance and commitment therapy; BMI = body mass index; CTQ = childhood trauma questionnaire (Bernstein et al., 1994 a, 1994b); CECA.Q = childhood experience of care and abuse questionnaire (Smith et al., 2002); CBT = cognitive behavioral therapy; CSA = childhood sexual abuse; DBT = Dialectical Behavior Therapy; EDE = eating disorder examination (Fairburn & Cooper, 1993); EDE-Q = eating disorder examination questionnaire (Fairburn, Cooper & O’Connor, 2008); EDI-2 = eating disorder inventory-2 (Garner, 1991); EPSI = eating pathology symptoms inventory (Forbush et al., 2013); ERP = exposure response and prevention; ICAT = integrated cognitive affective therapy; IPT = interpersonal psychotherapy; SIAB = structured inventory for anorexic and bulimic syndromes (Fichter et al., 1998); TEC = traumatic experiences checklist (Nijenhuis et al., 2002); OR = odds ratio; PCL-5 = post-traumatic stress disorder checklist for DSM-5 (Blevins et al., 2015); QWEP-5 = questionnaire on eating and weight patterns-5 (Yanovski et al., 2015); SCID-IV = structured clinical interview for DSM-IV (First et al., 1995); UTM = unified treatment model.

a Information provided by study author upon request.

As a result of the quality analysis, three studies were rated as “strong,” six were rated as “moderate,” and seven were rated as “weak.” Common methodological weaknesses were included using the unvalidated assessment tools (e.g., unstructured interviews assessing history of traumatic events), low rates of study completion (e.g., due to treatment dropout), and statistical analyses not controlling for potential confounds. The global quality analysis rating for each study is included in Table 1. Ratings for each category of the quality assessment tool can be found in the supplemental material.

### Effects on Eating Disorder Symptoms

#### Trauma history

Study findings and effect sizes are summarized in Table 2. Of the seven studies investigating the effect of trauma history on eating disorder symptoms as an outcome of treatment, four found that trauma history negatively affected eating disorder symptoms posttreatment (Cassioli et al., 2022; Fichter et al., 2008; Serra et al., 2020; Vrabel et al., 2010) and three found mixed effects (Anderson et al., 1997; Hazzard et al., 2021; Rienecke et al., 2022).

Of the four studies finding consistent significant effects, two measured history of childhood trauma (Cassioli et al., 2022; Vrabel et al., 2010), one examined any history of sexual abuse (Fichter et al., 2008), and the remaining study included all forms of trauma exposure (Serra et al., 2020). For individuals exposed to trauma in childhood, Cassioli et al. (2022) found that lower treatment efficacy for eating disorder symptoms at a 1-year follow-up, mediated by higher baseline emotion dysregulation, compared to those without a history of childhood trauma. Vrabel et al. (2010) similarly found that childhood sexual abuse was negatively associated with improvement in eating disorder symptoms at a 5-year follow-up. In the study measuring any history of severe and violent sexual abuse, this form of trauma exposure was found to predict a greater likelihood of continuing to meet eating disorder diagnostic criteria at a 12-year follow-up for BED, but had no impact on symptoms for participants with BN purging subtype (Fichter et al., 2008). Finally, one study found that although the number of (any) previous traumatic experiences did not predict reduction in binge eating during treatment, greater self-reported impact of these experiences was associated with lower likelihood of remission from BED at discharge (Serra et al., 2020).

Three studies reported mixed findings, of which two assessed for childhood trauma exposure (Hazzard et al., 2021; Rienecke et al., 2021) and one examined sexual abuse (Anderson et al., 1997). The first mixed findings for childhood trauma by Rienecke et al. (2022) found that it was positively associated with frequency of binge eating at discharge when aggregated across eating disorder diagnoses. However, when examining eating disorder diagnoses separately, they found that childhood trauma was associated with greater frequency of purging post-discharge for individuals with AN-R and BED, but not for AN-BP, BN, ARFID, or OSFED and with no significant effects for binge eating or restriction. The second mixed results for childhood trauma were found by Hazzard et al. (2021), who reported that childhood abuse was not associated with global eating disorder pathology at discharge; however, they found effects of childhood abuse on binge eating at follow-up when moderated by PTSD. The final mixed results were from Anderson et al. (1997), whose study found that a history of sexual abuse was associated with lower rates of abstinence from eating disorder behaviors at a 3-month follow-up and higher hospitalization rates between discharge and follow-up. However, they also found that sexual abuse did not affect the degree of change in a global measure of eating disorder symptoms from pretreatment to a 3-month follow-up.

#### Post-traumatic stress disorder

Of the four studies that investigated the impact of PTSD diagnosis or symptoms on eating disorder pathology, three found significant effects (Hazzard et al., 2021; Mensinger, 2021; Scharff et al., 2021) and one found no effect on eating disorder outcomes (Mitchell et al., 2021). Two studies with significant findings measured PTSD diagnosis and found that it was associated with more frequent binge eating at discharge when controlling for baseline binge eating (Hazzard et al., 2021) and predicted greater symptom recurrence from discharge to a 6-month follow-up (Scharff et al., 2021). Each of these following studies also found that PTSD acted as a moderator: In the study by Hazzard et al. (2021), PTSD moderated the effect of childhood abuse on binge eating at follow-up, such that a history of moderate or severe abuse predicted more frequent binge eating only for those with a diagnosis of PTSD. For Scharff et al. (2021), PTSD moderated change in eating disorder symptoms from admission to discharge, such that participants with a diagnosis of PTSD reported greater rates of symptom improvement, but then were also more likely to relapse in the 6 months posttreatment. The third study with significant findings by Mensinger (2021) measured “traumatic stress,” though the assessment tool used by this study was a measure of PTSD symptoms that was designed to align with DSM-5 diagnostic criteria for PTSD (Blevins et al., 2015). As such, it may be considered a continuous measure of PTSD symptoms rather than a categorical indicator of whether threshold diagnostic criteria were met. They found that reduced traumatic stress during treatment mediated improvement in eating concerns, binge eating, and overvaluation of weight/shape at discharge (Mensinger, 2021). These authors did not examine the effect of simply having high levels of traumatic stress at pretreatment on posttreatment outcomes. The final study on PTSD by Mitchell et al. (2021) found that PTSD diagnosis did not affect the trajectory of eating disorder symptom change or the likelihood of clinically significant symptom change from admission to discharge or discharge to a 6-month follow-up.

### Effects on Treatment Duration and Dropout

#### Trauma history

Of the four studies that assessed the relationship between trauma history and treatment dropout, three found that a history of childhood trauma experiences was associated with greater likelihood of treatment dropout (Mahon, Bradley, et al., 2001; Mahon, Winston, et al., 2001; Pingani et al., 2012) and one found mixed effects (Carter et al., 2006). Specifically, Pingani et al. (2012) found that experiences of childhood trauma were significantly more common in treatment dropouts than treatment completers. Two other studies found a dose–effect response, such that greater number of traumatic events in childhood predicted greater likelihood of treatment dropout (Mahon, Bradley, et al., 2001; Mahon, Winston, et al., 2001). The fourth study found that a history of childhood sexual abuse did not predict the mean time to discharge or overall premature dropout (Carter et al., 2006). However, when separated into AN subtypes, they found that childhood sexual abuse was a significant predictor of dropout for participants with AN binge-purge subtype but not those with AN restricting subtype.

#### Post-traumatic stress disorder

Two studies assessed the impact of PTSD on treatment dropout, with mixed findings. Mitchell et al. (2021) found that PTSD diagnosis was not associated with treatment dropout from a residential program. However, Trottier (2020) found that PTSD diagnosis predicted a 2.32-times greater likelihood of premature treatment termination from a hospital outpatient service.

### Effect of Trauma Exposure on Other Treatment Outcomes

There were two studies including additional eating disorder treatment outcomes, both assessing the effect of trauma history rather than PTSD. Castellini et al. (2020) found that history of childhood abuse was associated with greater likelihood of and shorter mean time to resumption of menses in females with AN. Carter et al. (2006) found that mean BMI at discharge did not significantly differ for those with and without a history of childhood sexual abuse.

## Discussion

This review has revealed the varied findings of current literature on how trauma and PTSD affect eating disorder treatment outcomes. Most previous studies have focused on eating disorder psychopathology and dropout as the outcomes of treatment. More than half of the studies included in this review indicated potentially detrimental effects of trauma and PTSD on whether participants complete and/or benefit from eating disorder treatment. Effect sizes varied, with just over half of the studies that included an effect size reporting small effects, but four studies reporting medium or large effect sizes (Anderson et al., 1997; Fichter et al., 2008; Hazzard et al., 2021; Trottier, 2020). However, the presence of mixed findings highlights the need for more detailed research for examining the impact for different diagnostic groups, treatment settings, and based on the way in which trauma and trauma-related symptoms such as PTSD is measured.

Most studies that measured eating disorder symptoms found that a history of trauma or PTSD diagnosis was associated with an adverse impact on posttreatment eating disorder psychopathology. Similarly, studies investigating premature treatment termination largely indicated that trauma and PTSD have negative consequences for treatment completion. There was some research to indicate that the effects may be conditional on eating disorder diagnosis (history of sexual abuse affecting AN-BP and BED, but not AN-R or BN-P [Carter et al., 2006; Fichter et al., 2008]). Other findings varied by timepoint, with PTSD predicting binge eating at end-of-treatment but history of childhood abuse predicted it at a 6-month follow-up (Hazzard et al., 2021). Finally, one study highlighted the perceived impact, rather than the number of traumatic experience(s) (Serra et al., 2020). Only one study examined BMI as the treatment outcome and found no effect of childhood sexual abuse (Carter et al., 2006). Overall, these findings are in support of the existing research on the detrimental effect of psychiatric comorbidities on eating disorder treatment outcomes (Fewell et al., 2017; Lydecker & Grilo, 2021; Vall & Wade, 2015).

Despite the potential significant impacts of trauma and PTSD for individuals with eating disorders, the results of this review nonetheless suggested that such individuals may still benefit from eating-disorder-specific treatment. This is exemplified in the two recent studies which found that PTSD and childhood abuse may be associated with a steeper rate of symptom change in eating pathology, including biological markers such as resumption of menstruation (Castellini et al., 2020; Scharff et al., 2021). In the study examining resumption of menses, the authors proposed that individuals with a history of abuse may experience a different trajectory of illness for their eating disorder, in which resumption of menses may not necessarily be a marker of recovery (Castellini et al., 2020). In the study measuring changes in global eating pathology, the authors suggested that their findings could be due to more severe baseline eating disorder pathology in cases of PTSD (Scharff et al., 2021). This indicates that the finding of larger improvements in eating disorder symptoms for individuals with PTSD may be due to regression to the mean, as such individuals typically begin treatment with more severe eating disorder psychopathology. These two findings highlight that although individuals who have experienced either traumatic events or PTSD may thus experience poorer eating disorder outcomes overall, they also have capacity to benefit from eating disorder treatment.

### Methodological Review

The quality analysis revealed several important limitations of the current literature on trauma, PTSD, and eating disorder treatment. Firstly, measures of trauma and PTSD were varied and often relied upon unstandardized semi-structured or unstructured interviews in which the interviewer determined whether a history of childhood abuse was present or the diagnostic criteria for PTSD were met (8 out of 16 studies). It is difficult to determine the validity of these assessment tools and whether the results of studies can be readily compared. In addition, only one study on childhood trauma exposure measured its perceived impact, rather than simply its occurrence. This may limit the findings of studies on trauma history, as not all individuals who go through traumatic events may experience an enduring impact; indeed, one recent review found that in children experiencing some form of traumatic event, the prevalence of PTSD was 21% 1 month post-trauma and further reduced to 11% 1 year post-event (Hiller et al., 2016). Similarly, a study with a community sample of adults found that 47% of the individuals had been exposed to traumatic events, but only 14.3% met the criteria for PTSD (White et al., 2015). This indicates the value of measuring not only the history of traumatic events, but also the perceived impact of these events and/or ongoing trauma-related symptoms.

Measurement issues also included how the eating disorder treatment outcome was measured. Many studies compared differences in levels of eating pathology solely at posttreatment or follow-up between participants with and without a history of trauma or PTSD, without examining change over time. It is difficult to know with this method of measuring treatment outcome whether any differences observed simply reflect the elevated baseline symptoms which are seen in individuals affected by trauma and PTSD (Scharff et al., 2019, 2021), and as such does not present a full picture of the response to treatment. In contrast, some studies did examine change in eating disorder psychopathology from pre to posttreatment (Anderson et al., 1997; Mitchell et al., 2021; Scharff et al., 2021). This method allows for a clearer understanding of treatment response relative to baseline pathology. However, as mentioned previously, their findings may also be limited by regression to the mean due to the higher baseline symptoms of traumatized individuals. Further understanding of how trauma and PTSD affect eating disorder treatment response will also require investigation of the form of treatment used. Although many studies included in this review described the use of specific evidence-based psychological therapies such as CBT, some provided more generic descriptions of “individual” or “group therapy.” It is unclear as to which extent all of the treatments used were evidence-based, and whether this might influence the degree to which trauma and PTSD affect treatment outcomes.

Another methodological limitation identified in the quality analysis was high rates of dropout in some studies. Dropout rates of greater than 20% were evident in five studies for which eating disorder-related symptoms were the treatment outcomes being assessed (Castellini et al., 2020; Hazzard et al., 2021; Mensinger, 2021; Rienecke et al., 2021; Scharff et al., 2021). In two of these studies, completion rates were less than 60% (Rienecke et al., 2021; Scharff et al., 2021). This limitation is not unique to studies of eating disorder treatment, as research on CBT and other treatments for depression have similarly found premature dropout rates of 20%−26% (Cooper & Conklin, 2015; Fernandez et al., 2015). However, attrition can reduce the internal and external validity of studies by skewing the characteristics of the sample so that they may lose representativeness of the population being studied (Crutzen et al., 2015; Nam & Toneatto, 2016). As such, it is unclear whether trauma or PTSD could have contributed to the high rates of dropout, which was found in other studies included in this review. Although a degree of attrition is likely to be unavoidable in any study of eating disorder treatment, researchers could aim to use appropriate methods of missing data imputation and to ascertain information from treatment non-completers about the reason for premature dropout (Eisner et al., 2019; Nam & Toneatto, 2016).

Finally, as with much research on eating disorder treatment, the studies included in this review were all from Western countries and largely included young cisgender female participants. Some ethnic minority groups, such as African Americans and First Nations peoples, have been found to experience higher rates of PTSD (Alegría et al., 2013; Nasir et al., 2021). Similarly, research indicates that transgender individuals experience higher rates of traumatic events and PTSD than cisgender populations (Livingston et al., 2022; Shipherd et al., 2011), including in a sample of individuals receiving residential eating disorder treatment (Brewerton et al., 2022). As such, future research on eating disorder treatment and trauma should aim to include a higher proportion of minority groups who may be at greater risk of experiencing detrimental effects of trauma and PTSD.

### Strengths and Limitations of This Review

There was a compelling rationale for this review, although considerable previous literature has examined the role of trauma and PTSD in eating disorder epidemiology, research on their relationship with treatment outcomes has been less studied, and to our knowledge there has been no previous systematic review of this topic. Other strengths included the use of multiple reviewers to assess the relevance of articles from the literature search and to apply the quality assessment tool. However, this review did not include grey literature or studies not available in English and relevant research from these sources may have been missed. As the studies included showed considerable heterogeneity in the treatment setting and approach, eating disorder presentations, and measure of trauma or PTSD, it was not possible to apply meta-analytic methods. This prevents conclusions about the impact of trauma and PTSD on eating disorder outcomes for specific diagnostic groups, treatment settings, or variations by type of traumatic event.

### Future Directions and Implications

The critical findings and implications of this review are summarized in Tables 3 and 4, respectively. Directions for future research should therefore include comparisons between eating disorder presentations, treatment settings, and exposure to different types of traumatic events. With regard to treatment settings, for example, it would be useful for future studies to examine whether individuals with a history of trauma exposure are more likely to benefit from residential or intensive outpatient treatment as opposed to hospital inpatient services, which can present a risk of re-traumatization due to restrictive practices such as involuntary admission, seclusion, restraint, and coercive refeeding (Muskett, 2014; Treasure et al., 2011). Research to date has also not examined the impact of complex CPTSD, a relatively recent diagnosis created to reflect the symptoms of prolonged, repeated trauma exposure such as forms of childhood abuse (Herman, 1992). The diagnostic profile of CPTSD has been found to be distinct from PTSD (Brewin et al., 2017; Karatzias et al., 2017) and may be particularly relevant to eating disorder populations, given the high prevalence of childhood abuse among such individuals (Rienecke et al., 2022). Future research should therefore seek to include CPTSD symptoms in analyses of the relationship between trauma effects and eating disorder treatment outcomes, and to compare its effects to those of PTSD. Finally, future studies could consider treatment outcomes other than those included in this review, such as how trauma and PTSD affect clinical impairment and quality of life following an eating disorder treatment.

**Table 3.** Critical Findings of the Current Review.

Critical Findings
Following eating disorder treatment, history of trauma and trauma-related symptoms may be associated with more severe eating disorder psychopathology.
Experiences of trauma and PTSD may be associated with greater likelihood of dropout from eating disorder treatment.
Individuals who have experienced trauma and PTSD still have capacity to benefit from treatment primarily for an eating disorder.

**Table 4.** Implications of the Current Review.

Domain	Implications
Practice	• Trauma and PTSD should be recognized as a potential indicator of poor prognosis in eating disorder treatment. • Eating disorder treatment services should consider implementing additional support for individuals with a history of trauma or PTSD so as to prevent dropout and increase likelihood of good treatment outcomes. • Individuals affected by trauma or PTSD should receive thorough discharge planning, such as transfer to a step-down in care, to support treatment gains and reduce risk of relapse. • Eating disorder clinicians should receive training in trauma-informed care and trauma-focused treatments.
Research	• More research is needed to compare how trauma and PTSD affect treatment outcomes for different eating disorder treatment settings, eating disorder presentations, and types of trauma. • Research could examine the potential benefits of combining eating disorder and trauma-focused treatment approaches.

The findings of this review have implications for treatment planning of those with both an eating disorder and a history of trauma exposure or lasting symptoms such as PTSD. The results suggest that such individuals may have a greater propensity to drop out from treatment, and could therefore benefit from closer clinical monitoring, increased treatment intensity, or involvement of additional supports such as family members. Previous research has suggested that eating disorder behaviors are employed as a way of coping with trauma symptoms (Trottier & MacDonald, 2017), and thus it may be the case that by reducing disordered eating, individuals are left more emotionally vulnerable to their trauma symptoms, which may partially explain the greater dropout rates as well. It is important to note that some studies have demonstrated that when they complete treatment, people with trauma exposure or PTSD can experience significant improvements in their eating disorder. However, given that one study found that these eating disorder improvements are directly related to the magnitude of improvements in trauma symptoms (Mensinger, 2021), may suggest that combined treatment approaches addressing both the effects of trauma and eating pathology may pay the greatest dividends. At discharge, individuals impacted by trauma or PTSD may continue to experience significant levels of eating disorder psychopathology despite improvements and therefore benefit from comprehensive discharge planning involving a step-down in care rather than stopping all treatment.

The effect of trauma and PTSD on eating disorder treatment outcomes highlights the importance of clinicians receiving training to understand, assess, and treat trauma in eating disorder populations. Brewerton (2018) argues for the importance of both trauma-informed care and trauma-focused treatments. SAMHSA’s (2014) model of trauma-informed care involves understanding effects of exposure to traumatic events, integrating this understanding into organizational policies, recognizing symptoms, avoiding re-traumatization, and adhering to key principles including safety, transparency, collaboration, and empowerment. Brewerton (2018) makes suggestions on how this model can be applied to the treatment of eating disorders, including informed consent around potentially distressing practices such as weighing and understanding and mitigating the potentially traumatizing effects of medical interventions such as nasogastric refeeding. Regarding trauma-focused treatments, clinicians may consider addressing PTSD symptoms either prior to or in parallel with the treatment of eating disorder behaviors, using evidence-based treatments for PTSD such as prolonged exposure or trauma-focused CBT (Watkins et al., 2018). A recent randomized controlled trial examined the effectiveness of cognitive processing therapy (CPT) integrated with CBT for eating disorders, following initial intensive eating disorder treatment (Trottier et al., 2022). They found that this integrated approach was effective in improving PTSD symptoms. Thus, eating disorder treatment settings should consider incorporating both principles of trauma-informed care and targeted trauma-focused treatments for individuals with relevant symptoms.

## Conclusion

This review has presented an overview of current literature for the effect of trauma and PTSD on eating disorder treatment outcomes. The results reveal that both a history of traumatic experiences and trauma-related symptoms such as those present in PTSD may be associated with more severe eating disorder pathology posttreatment and a greater likelihood of treatment non-completion. Further research into this area is needed to support these effects and to determine whether there are differences based on the type of traumatic event experienced, eating disorder presentation, or treatment setting. This research will be valuable in tailoring treatment and improving outcomes for the substantial number of individuals with eating disorders who have been affected by trauma and PTSD.

## Supplemental Material

sj-docx-1-tva-10.1177_15248380231167399 – Supplemental material for A Systematic Review of the Effect of PTSD and Trauma on Treatment Outcomes for Eating Disorders Supplemental material, sj-docx-1-tva-10.1177_15248380231167399 for A Systematic Review of the Effect of PTSD and Trauma on Treatment Outcomes for Eating Disorders by Sinead Day, Phillipa Hay, Wadad. Kathy Tannous, Scott J. Fatt and Deborah Mitchison in Trauma, Violence, & Abuse
