# Supplementary material for: A Systematic Review of the Effect of PTSD and Trauma on Treatment Outcomes for Eating Disorders
Source: Trauma Violence Abuse. 2023 Apr 26;25(2):947–64. doi: 10.1177/15248380231167399 (PMC10913314; doi:10.1177/15248380231167399)
Supplement: sj-docx-1-tva-10.1177_15248380231167399 – Supplemental material for A Systematic Review of the Effect of PTSD and Trauma on Treatment Outcomes for Eating Disorders [file sj-docx-1-tva-10.1177_15248380231167399.docx]

**Table 5.** *Quality assessment tool ratings for studies included in systematic review.*

| Study | Selection bias | Study design | Confounders | Blinding | Data collection methods: Trauma measure | Data collection methods: Treatment outcome measure | Withdrawals and drop-outs | Global rating |
| --- | --- | --- | --- | --- | --- | --- | --- | --- |
| Anderson et al. (1997) | Moderate | Moderate | Moderate | Moderate | Weak | Strong | Strong | Moderate |
| Carter et al. (2006) | Moderate | Moderate | Weak | Moderate | Weak | Weak | Moderate | Weak |
| Cassioli et al. (2022) | Moderate | Moderate | Moderate | Moderate | Strong | Strong | Strong | Strong |
| Castellini et al. (2020) | Moderate | Moderate | Moderate | Moderate | Weak | Strong | Moderate | Moderate |
| Fichter et al. (2008) | Moderate | Moderate | Weak | Moderate | Weak | Strong | Strong | Weak |
| Hazzard et al. (2021) | Weak | Strong | Strong | Moderate | Strong | Strong | Moderate | Moderate |
| Mahon, Bradley et al. (2001) | Weak | Moderate | Weak | Moderate | Weak | Strong | Moderate | Weak |
| Mahon, Winston et al. (2001) | Moderate | Moderate | Weak | Moderate | Weak | Strong | Moderate | Weak |
| Mensinger (2021) | Weak | Moderate | Moderate | Moderate | Strong | Strong | Weak | Weak |
| Mitchell et al. (2021) | Moderate | Moderate | Strong | Moderate | Weak | Strong | Weak | Weak |
| Pingani et al. (2012) | Moderate | Moderate | Weak | Moderate | Weak | Strong | Moderate | Weak |
| Rienecke et al. (2022) | Moderate | Moderate | Strong | Moderate | Strong | Strong | Weak | Moderate |
| Scharff et al. (2021) | Moderate | Moderate | Strong | Moderate | Moderate | Strong | Weak | Moderate |
| Serra et al. (2020) | Moderate | Moderate | Strong | Moderate | Moderate | Strong | Strong | Strong |
| Trottier (2020) | Moderate | Moderate | Strong | Moderate | Strong | Strong | Moderate | Strong |
| Vrabel et al. (2010) | Moderate | Moderate | Weak | Moderate | Moderate | Strong | Strong | Moderate |
